# 1,3-Bis[(6-methyl-2-pyrid­yl)meth­yl]imidazolium bromide

**DOI:** 10.1107/S1600536809006710

**Published:** 2009-02-28

**Authors:** Ga Young Kim, Sang-Kyu Park, Dong-Heon Lee, Gyungse Park

**Affiliations:** a Department of Chemistry, Chonbuk National University, Jeonju, Chonbuk 561-756, Republic of Korea; b Department of Chemistry, Kunsan National University, Kusan, Chonbuk 573-701, Republic of Korea

## Abstract

The title compound, C_17_H_19_N_4_^+^·Br^−^, is built up from 1,3-bis­[(6-methyl-2-pyridin­yl)meth­yl]imidazolium cations and bromide anions. Each of two 6-methyl-2-pyridyl rings is rotated out of the imidazole plane, making dihedral angles of 79.90 (9) and 86.40 (9)°. The packing is consolidated by aromatic π–π inter­actions between the pyridine rings of neighbouring mol­ecules [centroid–centroid distance = 3.554 (2) Å] and by weak C—H⋯N and C—H⋯Br hydrogen bonds.

## Related literature

For the synthesis of *N*-heterocyclic carbenes, see: Arduengo *et al.* (1991 ▶); Enders *et al.* (1996 ▶); Frenzel *et al.* (1999 ▶); Gardiner *et al.* (1999 ▶); Herrmann *et al.* (1998 ▶); McGuinness *et al.* (1998 ▶); Öfele (1968 ▶); Wanzlick & Schonherr (1968); Wanzlick & Schönherr (1968 ▶); Zhang & Trudell (2000 ▶). For related structures, see: Weskamp *et al.* (1999*a* ▶ *b* ▶).
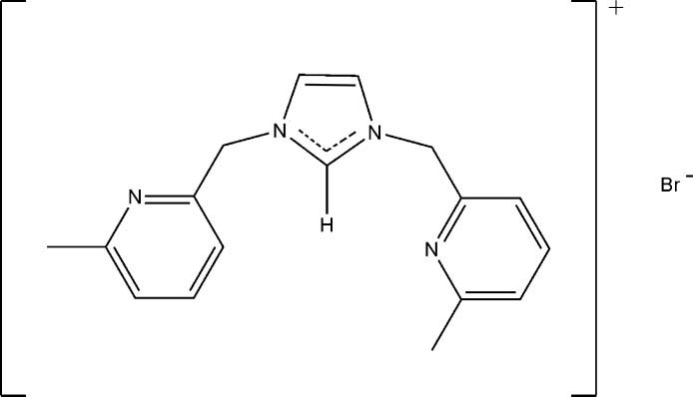


## Experimental

### 

#### Crystal data

C_17_H_19_N_4_^+^·Br^−^
*M* *_r_* = 359.27
Monoclinic, 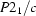

*a* = 8.2951 (4) Å
*b* = 12.4992 (5) Å
*c* = 16.1786 (7) Å
β = 95.709 (1)°
*V* = 1669.11 (13) Å^3^
*Z* = 4
Mo *K*α radiation
μ = 2.47 mm^−1^
*T* = 173 K
0.40 × 0.25 × 0.15 mm

#### Data collection

Bruker SMART CCD area-detector diffractometer
Absorption correction: multi-scan (*SADABS*; Sheldrick, 1996 ▶) *T*_min_ = 0.439, *T*_max_ = 0.709
10482 measured reflections
3923 independent reflections
2861 reflections with *I* > 2σ(*I*)
*R*_int_ = 0.070

#### Refinement

*R*[*F* ^2^ > 2σ(*F* ^2^)] = 0.037
*wR*(*F* ^2^) = 0.094
*S* = 1.00
3923 reflections
201 parameters
H-atom parameters constrained
Δρ_max_ = 0.92 e Å^−3^
Δρ_min_ = −0.50 e Å^−3^

### 

Data collection: *SMART* (Bruker, 1997 ▶); cell refinement: *SAINT* (Bruker, 1997 ▶); data reduction: *SAINT*; program(s) used to solve structure: *SHELXS97* (Sheldrick, 2008 ▶); program(s) used to refine structure: *SHELXL97* (Sheldrick, 2008 ▶); molecular graphics: *SHELXTL* (Sheldrick, 2008 ▶); software used to prepare material for publication: *SHELXTL*.

## Supplementary Material

Crystal structure: contains datablocks I, global. DOI: 10.1107/S1600536809006710/lx2088sup1.cif

Structure factors: contains datablocks I. DOI: 10.1107/S1600536809006710/lx2088Isup2.hkl

Additional supplementary materials:  crystallographic information; 3D view; checkCIF report

## Figures and Tables

**Table 1:** Hydrogen-bond geometry (Å, °)

*D*—H⋯*A*	*D*—H	H⋯*A*	*D*⋯*A*	*D*—H⋯*A*
C7—H7*A*⋯N4^i^	0.99	2.34	3.308 (4)	165
C7—H7*B*⋯Br^ii^	0.99	2.79	3.660 (3)	147
C9—H9*A*⋯Br	0.95	2.91	3.695 (3)	141
C10—H10*A*⋯Br^i^	0.95	2.68	3.521 (3)	148
C11—H11*A*⋯Br	0.99	2.93	3.801 (3)	147
C11—H11*B*⋯Br^i^	0.99	2.89	3.690 (3)	138

Symmetry codes: (i) 
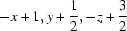
; (ii) 
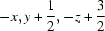
.

**Fig. 1.**
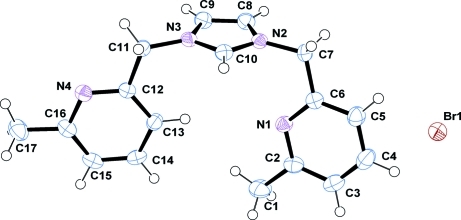
The molecular structure of the title compound, showing displacement ellipsoids drawn at the 50% probability level.

**Fig. 2.**
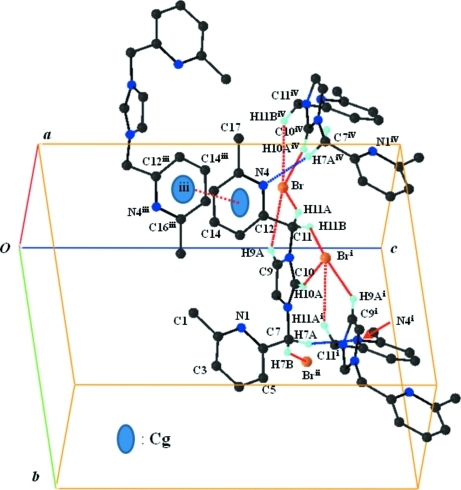
π—π, C—H···N and C—H···Br interactions (dotted lines) in the title compound. Cg denotes the ring centroids. [Symmetry code : (i) -x+1, y+1/2, -z+3/2; (ii) -x, y+1/2, -z+3/2; (iii) -x+1, -y, -z+1; (iv) -x+1, y-1/2, -z+3/2.]

## supplementary crystallographic information

### Comment

Many intensive researches have been focused on the synthesis of *N*-heterocyclic carbenes (NHCs) ligands due to their potential applications in important organic syntheses, such as Pd–catalysed Heck– and Suzuki–coupling, Co–catalysed ethylene copolymerisation, Ru–catalysed olefin metathesis and Rh–catalyse hydrosilylation (Frenzel *et al.*, 1999; Enders *et al.*, 1996; Gardiner *et al.*, 1999; McGuinness *et al.*, 1998; Weskamp *et al.*, 1999*a*,*b*); Zhang & Trudell, 2000). We have interested in the use of tridentate *N*-heterocyclic carbene ligands. Here we report the crystal structure of the title compound, 1,3-bis[(6-methyl-2-pyridinyl)methyl]imidazolium bromide (Fig. 1).

The asymmetric unit of the title compound  consists the C_17_H_9_N_4_ cation and Br anion. Each of two 6–methylpyridine rings is rotated out of the imidazole plane, with dihedral angle of N1/C2–C6 of 79.90 (9)° and N4/C12–C16 of 86.40 (9)°, respectively. The crystal packing (Fig. 2) is stabilized by intermolecular aromatic π—π interactions between the pyridine rings of neighbouring molecules. The Cg—Cg^iii^ distance of 3.554 (2) Å (Cg is the centroid of the N1/C2-C6 pyridine ring; symmetry code as in Fig. 2). The molecular packing is further stabilized by C—H···N interactions between the hydrogen of 7–methylene group and the N atom of pyridine ring of the neighbouring molecule, with a C7—H7A···N4^i^ separation of 2.34 (1) Å (Table 1 and Fig. 2; symmetry code as in Fig. 2). Additionally, five different intermolecular C—H···Br hydrogen bonds in the structure are observed (Table 1 & Fig. 2).

### Experimental

Synthesis of H(MepyCH_2_)Im (1a): A mixture of imidazole (0.36 g, 6.0 mmol), 2-bromomethyl-6-methylpyridine (1.11 g, 6.0 mmol) and triethylamine (1.82 g, 1.8 mmol) in toluene (50 mL) was refluxed at 383 K for 10 h. After cooling, saturated aqueous NaHCO_3_ solution was added and extracted with CH_2_Cl_2_ (3 x 20 mL). The combined organic fractions were dried over Na_2_SO_4_, filtered, and concentrated under reduced pressure to afford a dark colored solid in 53.0 % yield. Spectroscopic analysis: ^1^H NMR(CDCl_3_, 400MHz) : δ 7.62 (s, 1H, CH), 7.54 (t, 1H, J = 10 Hz, CH), 7.12 (s, 1H, CH), 7.09 (d, 1H, J = 1 Hz, CH), 6.99 (s, 1H, CH), 6.70 (d, 1H, J = 0.6Hz, CH), 5.21(s, 2H, CH_2_), 2.56 (s, 3H, CH_3_). ^13^C NMR (CDCl_3_, 100MHz): δ 157.8, 154.1, 137.5, 136.6, 123.2, 122.5, 121.4, 118.9, 52.3, 23.9.

Synthesis of [H(MepyCH_2_)_2_-Im]Br (1): A mixture of (1a), H(MepyCH_2_)Im, (0.52 g, 3.0 mmol) and 2-bromomethyl-6-methylpyridine (0.56 g, 3.0 mmol) in toluene (50 mL) was refluxed at 383 K for 14 h. After cooling, the solvents were removed by high-vacuum rotary evaporation. The residue was washed with Et_2_O (5 x100 mL), and dried under the reduced pressure to afford a brown solid in 89.0 % yield. Single crystlas suitable for X-ray crystallography were obtained by Et_2_O diffusion into a MeOH solution of the compound. Spectroscopic analysis: ^1^H NMR (CDCl_3_, 400 MHz) : δ 10.52 (s, 1H, CH), 7.66 (s, 2H, CH), 7.62 (t, 2H, J = 7.8 Hz), 7.54 (d, 2H, J = 7 Hz, CH), 7.14 (d, 2H, J = 7 Hz, CH), 5.65 (s, 4H, CH_2_), 2.50 (s, 6H, CH_3_). ^13^C NMR (CDCl_3_, 100 MHz): δ 158.8, 151.3, 137.9, 137.3, 123.7, 122.1, 121.0, 54.2, 24.4.

### Refinement

All H atoms were geometrically positioned and refined using a riding model, with C—H = 0.95 Å for the aryl, 0.99 Å for the methylene, and 0.00 Å for the methyl H atoms, respectively, and with Uiso(H) = 1.2Ueq(C) for the aryl and methylene H atoms, and 1.5Ueq(C) for the methyl H atoms.

### Crystal data

**Table:**

C_17_H_19_N_4_^+^·Br^−^	*F*(000) = 736
*M**_r_* = 359.27	*D*_x_ = 1.430 Mg m^−^^3^
Monoclinic, *P*2_1_/*c*	Mo *K*α radiation, λ = 0.71073 Å
Hall symbol: -P 2ybc	Cell parameters from 3923 reflections
*a* = 8.2951 (4) Å	θ = 2.1–28.3°
*b* = 12.4992 (5) Å	µ = 2.47 mm^−^^1^
*c* = 16.1786 (7) Å	*T* = 173 K
β = 95.709 (1)°	Block, yellow
*V* = 1669.11 (13) Å^3^	0.40 × 0.25 × 0.15 mm
*Z* = 4	

### Data collection

**Table:**

Bruker SMART CCD area-detector diffractometer	3923 independent reflections
Radiation source: fine-focus sealed tube	2861 reflections with *I* > 2σ(*I*)
graphite	*R*_int_ = 0.070
Detector resolution: 10.0 pixels mm^-1^	θ_max_ = 28.3°, θ_min_ = 2.1°
φ and ω scans	*h* = −10→9
Absorption correction: multi-scan (*SADABS*; Sheldrick, 1996)	*k* = −8→16
*T*_min_ = 0.439, *T*_max_ = 0.709	*l* = −21→20
10482 measured reflections	

### Refinement

**Table:**

Refinement on *F*^2^	Primary atom site location: structure-invariant direct methods
Least-squares matrix: full	Secondary atom site location: difference Fourier map
*R*[*F*^2^ > 2σ(*F*^2^)] = 0.037	Hydrogen site location: difference Fourier map
*wR*(*F*^2^) = 0.094	H-atom parameters constrained
*S* = 1.00	*w* = 1/[σ^2^(*F*_o_^2^) + (0.0442*P*)^2^] where *P* = (*F*_o_^2^ + 2*F*_c_^2^)/3
3923 reflections	(Δ/σ)_max_ < 0.001
201 parameters	Δρ_max_ = 0.92 e Å^−^^3^
0 restraints	Δρ_min_ = −0.49 e Å^−^^3^

### Special details

**Table:**

Geometry. All esds (except the esd in the dihedral angle between two l.s. planes) are estimated using the full covariance matrix. The cell esds are taken into account individually in the estimation of esds in distances, angles and torsion angles; correlations between esds in cell parameters are only used when they are defined by crystal symmetry. An approximate (isotropic) treatment of cell esds is used for estimating esds involving l.s. planes.
Refinement. Refinement of F^2^ against ALL reflections. The weighted R-factor wR and goodness of fit S are based on F^2^, conventional R-factors R are based on F, with F set to zero for negative F^2^. The threshold expression of F^2^ > 2sigma(F^2^) is used only for calculating R-factors(gt) etc. and is not relevant to the choice of reflections for refinement. R-factors based on F^2^ are statistically about twice as large as those based on F, and R- factors based on ALL data will be even larger.

### Fractional atomic coordinates and isotropic or equivalent isotropic displacement parameters (Å^2^)

**Table:**

	*x*	*y*	*z*	*U*_iso_*/*U*_eq_	
Br	0.25933 (3)	−0.13979 (2)	0.728857 (17)	0.02867 (10)	
N1	0.2300 (3)	0.43039 (18)	0.55743 (13)	0.0278 (5)	
N2	0.1795 (3)	0.32251 (18)	0.69967 (13)	0.0249 (5)	
N3	0.3401 (3)	0.18620 (18)	0.70972 (12)	0.0251 (5)	
N4	0.6655 (3)	0.02641 (18)	0.63640 (13)	0.0278 (5)	
C1	0.3308 (4)	0.4122 (3)	0.42228 (19)	0.0461 (8)	
H1A	0.4447	0.3965	0.4401	0.069*	
H1B	0.3231	0.4511	0.3695	0.069*	
H1C	0.2699	0.3451	0.4151	0.069*	
C2	0.2610 (3)	0.4796 (2)	0.48712 (16)	0.0316 (6)	
C3	0.2275 (4)	0.5877 (2)	0.47302 (19)	0.0372 (7)	
H3A	0.2493	0.6205	0.4223	0.045*	
C4	0.1625 (4)	0.6461 (2)	0.5333 (2)	0.0405 (7)	
H4A	0.1389	0.7199	0.5249	0.049*	
C5	0.1314 (3)	0.5967 (2)	0.60658 (19)	0.0343 (7)	
H5A	0.0881	0.6358	0.6496	0.041*	
C6	0.1653 (3)	0.4883 (2)	0.61548 (16)	0.0268 (6)	
C7	0.1255 (3)	0.4340 (2)	0.69380 (17)	0.0283 (6)	
H7A	0.1770	0.4740	0.7422	0.034*	
H7B	0.0068	0.4363	0.6963	0.034*	
C8	0.0851 (3)	0.2329 (2)	0.68219 (17)	0.0297 (6)	
H8A	−0.0289	0.2316	0.6684	0.036*	
C9	0.1843 (3)	0.1478 (2)	0.68830 (17)	0.0302 (6)	
H9A	0.1538	0.0750	0.6796	0.036*	
C10	0.3325 (3)	0.2922 (2)	0.71530 (15)	0.0257 (6)	
H10A	0.4222	0.3386	0.7283	0.031*	
C11	0.4884 (3)	0.1223 (2)	0.71751 (17)	0.0280 (6)	
H11A	0.4651	0.0515	0.7411	0.034*	
H11B	0.5704	0.1582	0.7567	0.034*	
C12	0.5575 (3)	0.1063 (2)	0.63500 (16)	0.0251 (6)	
C13	0.5148 (3)	0.1686 (2)	0.56543 (16)	0.0298 (6)	
H13A	0.4382	0.2250	0.5667	0.036*	
C14	0.5875 (3)	0.1459 (2)	0.49366 (18)	0.0330 (6)	
H14A	0.5608	0.1865	0.4446	0.040*	
C15	0.6987 (3)	0.0642 (2)	0.49443 (17)	0.0331 (7)	
H15A	0.7500	0.0480	0.4460	0.040*	
C16	0.7350 (3)	0.0057 (2)	0.56687 (17)	0.0305 (6)	
C17	0.8531 (4)	−0.0873 (3)	0.5706 (2)	0.0445 (8)	
H17A	0.9412	−0.0744	0.6144	0.067*	
H17B	0.8980	−0.0943	0.5170	0.067*	
H17C	0.7964	−0.1535	0.5826	0.067*	

### Atomic displacement parameters (Å^2^)

**Table:**

	*U*^11^	*U*^22^	*U*^33^	*U*^12^	*U*^13^	*U*^23^
Br	0.02798 (15)	0.02717 (15)	0.03144 (16)	0.00081 (11)	0.00575 (11)	0.00151 (12)
N1	0.0321 (12)	0.0264 (12)	0.0249 (11)	−0.0012 (10)	0.0022 (10)	−0.0001 (9)
N2	0.0292 (12)	0.0238 (11)	0.0223 (11)	−0.0008 (9)	0.0057 (9)	−0.0003 (9)
N3	0.0275 (12)	0.0278 (12)	0.0205 (11)	0.0002 (10)	0.0043 (9)	0.0004 (9)
N4	0.0261 (12)	0.0312 (13)	0.0260 (11)	−0.0004 (10)	0.0014 (9)	−0.0010 (10)
C1	0.063 (2)	0.051 (2)	0.0266 (15)	0.0014 (17)	0.0125 (15)	0.0030 (14)
C2	0.0321 (15)	0.0388 (16)	0.0234 (13)	−0.0044 (13)	−0.0005 (11)	0.0019 (12)
C3	0.0381 (17)	0.0395 (18)	0.0332 (16)	−0.0073 (14)	−0.0008 (13)	0.0111 (14)
C4	0.0411 (17)	0.0278 (16)	0.052 (2)	0.0000 (13)	0.0002 (15)	0.0080 (14)
C5	0.0347 (16)	0.0268 (15)	0.0415 (17)	−0.0018 (13)	0.0035 (13)	−0.0015 (13)
C6	0.0234 (13)	0.0292 (15)	0.0273 (13)	−0.0039 (11)	−0.0008 (11)	−0.0005 (11)
C7	0.0329 (15)	0.0234 (14)	0.0290 (14)	0.0036 (11)	0.0057 (12)	−0.0016 (11)
C8	0.0279 (14)	0.0296 (15)	0.0318 (15)	−0.0043 (12)	0.0039 (12)	−0.0017 (12)
C9	0.0315 (14)	0.0280 (15)	0.0315 (14)	−0.0038 (12)	0.0052 (12)	−0.0033 (12)
C10	0.0315 (14)	0.0246 (14)	0.0211 (13)	−0.0041 (11)	0.0035 (11)	−0.0027 (10)
C11	0.0304 (14)	0.0296 (15)	0.0241 (13)	0.0062 (11)	0.0034 (11)	0.0028 (11)
C12	0.0241 (13)	0.0259 (13)	0.0251 (13)	−0.0039 (11)	0.0021 (11)	−0.0023 (11)
C13	0.0367 (16)	0.0260 (14)	0.0271 (14)	0.0015 (12)	0.0045 (12)	0.0020 (11)
C14	0.0381 (16)	0.0355 (16)	0.0258 (14)	−0.0051 (13)	0.0048 (12)	0.0055 (12)
C15	0.0308 (15)	0.0418 (17)	0.0283 (14)	−0.0083 (13)	0.0116 (12)	−0.0060 (13)
C16	0.0252 (14)	0.0361 (16)	0.0303 (14)	−0.0014 (12)	0.0023 (11)	−0.0053 (12)
C17	0.0432 (18)	0.053 (2)	0.0372 (17)	0.0125 (16)	0.0055 (14)	−0.0081 (15)

### Geometric parameters (Å, °)

**Table:**

N1—C6	1.339 (3)	C6—C7	1.503 (4)
N1—C2	1.341 (3)	C7—H7A	0.9900
N2—C10	1.324 (3)	C7—H7B	0.9900
N2—C8	1.380 (3)	C8—C9	1.343 (4)
N2—C7	1.464 (3)	C8—H8A	0.9500
N3—C10	1.330 (3)	C9—H9A	0.9500
N3—C9	1.389 (3)	C10—H10A	0.9500
N3—C11	1.462 (3)	C11—C12	1.518 (4)
N4—C16	1.339 (3)	C11—H11A	0.9900
N4—C12	1.341 (3)	C11—H11B	0.9900
C1—C2	1.506 (4)	C12—C13	1.385 (4)
C1—H1A	0.9800	C13—C14	1.390 (4)
C1—H1B	0.9800	C13—H13A	0.9500
C1—H1C	0.9800	C14—C15	1.375 (4)
C2—C3	1.393 (4)	C14—H14A	0.9500
C3—C4	1.371 (5)	C15—C16	1.389 (4)
C3—H3A	0.9500	C15—H15A	0.9500
C4—C5	1.383 (4)	C16—C17	1.518 (4)
C4—H4A	0.9500	C17—H17A	0.9800
C5—C6	1.388 (4)	C17—H17B	0.9800
C5—H5A	0.9500	C17—H17C	0.9800
			
C6—N1—C2	118.1 (2)	C9—C8—H8A	126.4
C10—N2—C8	108.7 (2)	N2—C8—H8A	126.4
C10—N2—C7	124.5 (2)	C8—C9—N3	107.0 (2)
C8—N2—C7	126.5 (2)	C8—C9—H9A	126.5
C10—N3—C9	108.2 (2)	N3—C9—H9A	126.5
C10—N3—C11	125.7 (2)	N2—C10—N3	108.8 (2)
C9—N3—C11	125.9 (2)	N2—C10—H10A	125.6
C16—N4—C12	118.2 (2)	N3—C10—H10A	125.6
C2—C1—H1A	109.5	N3—C11—C12	112.6 (2)
C2—C1—H1B	109.5	N3—C11—H11A	109.1
H1A—C1—H1B	109.5	C12—C11—H11A	109.1
C2—C1—H1C	109.5	N3—C11—H11B	109.1
H1A—C1—H1C	109.5	C12—C11—H11B	109.1
H1B—C1—H1C	109.5	H11A—C11—H11B	107.8
N1—C2—C3	122.1 (3)	N4—C12—C13	123.3 (2)
N1—C2—C1	117.0 (3)	N4—C12—C11	113.2 (2)
C3—C2—C1	120.9 (3)	C13—C12—C11	123.5 (2)
C4—C3—C2	119.1 (3)	C12—C13—C14	117.8 (3)
C4—C3—H3A	120.5	C12—C13—H13A	121.1
C2—C3—H3A	120.5	C14—C13—H13A	121.1
C3—C4—C5	119.5 (3)	C15—C14—C13	119.4 (3)
C3—C4—H4A	120.3	C15—C14—H14A	120.3
C5—C4—H4A	120.3	C13—C14—H14A	120.3
C4—C5—C6	118.1 (3)	C14—C15—C16	119.2 (3)
C4—C5—H5A	120.9	C14—C15—H15A	120.4
C6—C5—H5A	120.9	C16—C15—H15A	120.4
N1—C6—C5	123.1 (3)	N4—C16—C15	122.1 (3)
N1—C6—C7	118.9 (2)	N4—C16—C17	116.4 (3)
C5—C6—C7	118.0 (2)	C15—C16—C17	121.4 (3)
N2—C7—C6	113.2 (2)	C16—C17—H17A	109.5
N2—C7—H7A	108.9	C16—C17—H17B	109.5
C6—C7—H7A	108.9	H17A—C17—H17B	109.5
N2—C7—H7B	108.9	C16—C17—H17C	109.5
C6—C7—H7B	108.9	H17A—C17—H17C	109.5
H7A—C7—H7B	107.8	H17B—C17—H17C	109.5
C9—C8—N2	107.3 (2)		
			
C6—N1—C2—C3	−0.3 (4)	C8—N2—C10—N3	−0.9 (3)
C6—N1—C2—C1	−178.9 (3)	C7—N2—C10—N3	−175.1 (2)
N1—C2—C3—C4	0.8 (4)	C9—N3—C10—N2	0.9 (3)
C1—C2—C3—C4	179.4 (3)	C11—N3—C10—N2	175.9 (2)
C2—C3—C4—C5	−0.1 (4)	C10—N3—C11—C12	−89.3 (3)
C3—C4—C5—C6	−1.1 (4)	C9—N3—C11—C12	84.9 (3)
C2—N1—C6—C5	−1.0 (4)	C16—N4—C12—C13	−0.2 (4)
C2—N1—C6—C7	178.4 (2)	C16—N4—C12—C11	−179.6 (2)
C4—C5—C6—N1	1.7 (4)	N3—C11—C12—N4	−163.2 (2)
C4—C5—C6—C7	−177.7 (3)	N3—C11—C12—C13	17.3 (4)
C10—N2—C7—C6	73.2 (3)	N4—C12—C13—C14	0.4 (4)
C8—N2—C7—C6	−100.0 (3)	C11—C12—C13—C14	179.8 (3)
N1—C6—C7—N2	5.8 (3)	C12—C13—C14—C15	−0.5 (4)
C5—C6—C7—N2	−174.8 (2)	C13—C14—C15—C16	0.4 (4)
C10—N2—C8—C9	0.5 (3)	C12—N4—C16—C15	0.1 (4)
C7—N2—C8—C9	174.6 (2)	C12—N4—C16—C17	−178.4 (2)
N2—C8—C9—N3	0.1 (3)	C14—C15—C16—N4	−0.2 (4)
C10—N3—C9—C8	−0.6 (3)	C14—C15—C16—C17	178.2 (3)
C11—N3—C9—C8	−175.6 (2)		

### Hydrogen-bond geometry (Å, °)

**Table:**

*D*—H···*A*	*D*—H	H···*A*	*D*···*A*	*D*—H···*A*
C7—H7A···N4^i^	0.99	2.34	3.308 (4)	165
C7—H7B···Br^ii^	0.99	2.79	3.660 (3)	147
C9—H9A···Br	0.95	2.91	3.695 (3)	141
C10—H10A···Br^i^	0.95	2.68	3.521 (3)	148
C11—H11A···Br	0.99	2.93	3.801 (3)	147
C11—H11B···Br^i^	0.99	2.89	3.690 (3)	138

Symmetry codes: (i) −*x*+1, *y*+1/2, −*z*+3/2; (ii) −*x*, *y*+1/2, −*z*+3/2.
